# Emerging Therapies for Palmoplantar Pustulosis with a Focus on IL-23 Inhibitors

**DOI:** 10.3390/jcm14103273

**Published:** 2025-05-08

**Authors:** Kyung-Hwa Nam, Yoon-Seob Kim

**Affiliations:** 1 Department of Dermatology, Jeonbuk National University Medical School, Jeonju 54907, Republic of Korea; 2 Research Institute of Clinical Medicine of Jeonbuk National University, Biomedical Research Institute of Jeonbuk National University Hospital, Jeonju 54907, Republic of Korea; 3 Department of Dermatology, Bucheon St. Mary’s Hospital, College of Medicine, The Catholic University of Korea, Seoul 06591, Republic of Korea

**Keywords:** Palmoplantar pustulosis, IL-23 inhibitors, guselkumab, risankizumab, small-molecule inhibitors, apremilast, clinical trials

## Abstract

Palmoplantar pustulosis (PPP) is a chronic inflammatory skin disease characterized by recurrent pustules, erythema, and scaling on the palms and soles, leading to a significantly reduced quality of life. Although PPP shares some immunopathological features with psoriasis vulgaris, it is distinguished by unique genetic predispositions, including a higher prevalence in East Asian populations, and a complex immune profile, particularly dysregulation of the IL-23/Th17 axis and IL-36 cytokines. Recent advances in psoriasis treatment have highlighted IL-23 inhibitors, which target the p19 subunit to suppress Th17 activation and inflammatory cytokines. Clinical trials show that IL-23 inhibitors significantly improve disease severity and patient-reported outcomes in PPP while maintaining favorable safety profiles. Notably, guselkumab and risankizumab have recently been approved for PPP treatment in Japan and Korea. In contrast, IL-17 inhibitors and IL-36 blockers have yielded mixed results. A recent phase 3 trial in Japan demonstrated the significant efficacy of apremilast in treating PPP, with a favorable safety profile, suggesting that apremilast may be a promising treatment option for PPP. Due to PPP’s lower prevalence compared with psoriasis vulgaris, clinical trials remain limited. Further large-scale, controlled studies are needed to clarify the efficacy and long-term safety of these therapies in diverse populations. This review summarizes emerging evidence on IL-23 inhibitors and other treatments for PPP, detailing their mechanisms of action, clinical efficacy, safety profiles, current challenges, and future perspectives in optimizing therapy.

## 1. Introduction

Palmoplantar pustulosis (PPP) is a chronic, relapsing inflammatory disorder and the most common variant of pustular psoriasis. It is characterized by recurrent pustules, erythema, and scaling confined to the palms and soles. Although the condition is localized, it significantly impairs quality of life due to debilitating pain, pruritus, and disfiguring lesions in areas critical for daily function and social interaction [1,2,3,4]. While the classification of PPP as a variant of psoriasis versus a distinct clinical entity remains under debate, recent studies support its recognition as a separate disease with unique genetic and immunological features [5,6,7,8,9]. In contrast to psoriasis vulgaris, PPP predominantly affects middle-aged women and is strongly associated with smoking and certain infections (e.g., tonsillitis and dental infections), and drugs (e.g., TNF-a inhibitors) [10]. Similarly to psoriasis vulgaris, it is also linked with comorbidities such as psychological stress, metabolic syndrome, and cardiovascular and autoimmune diseases [4,11]. Moreover, the higher prevalence of PPP in East Asian populations (e.g., Japan, 0.12%) compared to Western populations (0.01–0.05%) suggests that genetic predispositions, environmental exposures, and lifestyle factors may contribute to this disparity [8,12].

The pathogenesis of PPP is multifactorial, involving a complex interplay between genetic susceptibility and environmental triggers. Central to its immunopathology is the dysregulation of cytokine networks—particularly the IL-23/IL-17 axis and IL-36 cytokines—that promote keratinocyte hyperproliferation and neutrophil recruitment. Conventional treatments, including acitretin, cyclosporine, phototherapy, and topical corticosteroids and vitamin D analogues, have been shown to have limited long-term efficacy and safety [13]. In contrast, recent biologic therapies targeting specific cytokines have transformed psoriasis management. Notably, IL-23 inhibitors, which selectively target the p19 subunit, have shown significant efficacy and safety in psoriasis vulgaris [14,15]. Emerging data from pivotal clinical trials also indicate that IL-23 inhibitors may offer a promising therapeutic option for PPP, although the current studies are limited by small sample sizes and short follow-up durations [16,17]. Based on these pivotal clinical trials, guselkumab and risankizumab have been approved for the treatment of moderate-to-severe PPP in Japan and Korea.

Acrodermatitis continua of Hallopeau is variably regarded as either a subtype of PPP or a distinct entity within the pustular psoriasis spectrum. It primarily affects the fingertips and periungual regions, typically presenting with sterile pustules, nail dystrophy, and chronic inflammation. However, due to limited clinical and therapeutic data on ACH, this review focuses on PPP, while acknowledging the clinical heterogeneity across its subtypes.

This review aims to summarize emerging evidence on IL-23 inhibitors in PPP by detailing their mechanisms of action, clinical efficacy, and safety profiles. In addition, it integrates insights on other biologics and small-molecule inhibitors to address current challenges and future perspectives in optimizing PPP therapy. Unlike earlier reviews that broadly examined the role of biologics in PPP [18,19,20], this review focuses specifically on IL-23 inhibitors to provide detailed clinical insights into these approved agents.

## 2. Pathophysiology of PPP

Due to the relatively limited number of studies on PPP, its pathophysiology remains less understood than that of psoriasis vulgaris. PPP is a complex inflammatory skin condition driven by an interplay of genetic predisposition, environmental triggers, and immune dysregulation. Although it shares certain pathogenic mechanisms with psoriasis vulgaris, PPP exhibits distinct features that necessitate tailored therapeutic approaches.

A genome-wide meta-analysis demonstrated that PPP is not associated with the genetic determinants commonly seen in psoriasis vulgaris. Instead, significant associations were identified with the *FCGR3A*/*FCGR3B* and *CCHCR1* loci, as well as the *IL4*/*IL13* interval, suggesting a notable genetic correlation between PPP and Th2-mediated diseases [8]. Moreover, Mendelian randomization analyses support a causal role for cigarette smoking in PPP [8].

The IL-23/IL-17 axis is a critical driver of inflammation in PPP. IL-23 promotes the differentiation and maintenance of Th17 cells, which secrete cytokines such as IL-17A, IL-17F, and IL-22. Elevated levels of these cytokines in PPP lesions stimulate keratinocyte hyperproliferation and amplify inflammation, contributing to pustule formation and erythematous plaques [21,22,23,24]. Recent single-cell RNA sequencing studies have also shown that memory CD4^+^ T cells in PPP lesions are skewed toward a Th17 phenotype, with a subset exhibiting both Th17 and Th2 markers—suggesting potential Th17-to-Th2 plasticity [25].

In addition, IL-36 cytokines—members of the IL-1 family—play an important role in PPP. IL-36α, IL-36β, and IL-36γ activate downstream NF-κB and MAPK pathways, leading to the production of pro-inflammatory chemokines and cytokines that recruit neutrophils and exacerbate inflammation [26]. IL36RN mutations, recognized as a key factor in familial or sporadic generalized pustular psoriasis, have also been identified in some patients with sporadic PPP, implicating the IL-36 pathway in its pathogenesis [27,28,29,30]. Furthermore, while IL-36 signaling—strongly induced by TNF-α and IL-17—has been established as a driver of plaque psoriasis, its inhibition has been shown to reduce inflammation and Th17 cell activity, potentially contributing to the unique pustule formation observed in PPP [31,32,33,34]. Notably, PPP lesions display increased expression of IL-1 and IL-36 family cytokines, along with neutrophil chemokines, but lower levels of Th1/Th17-associated markers compared to psoriasis vulgaris [35]. Recent studies have also highlighted the pathogenic role of IL-8 and neutrophil in the pathogenesis of PPP, especially for pustule formation in PPP [36,37,38].

Other genetic factors also contribute to PPP susceptibility. Mutations in *CARD14*, which mediates NF-κB signaling, have been associated with both gain- and loss-of-function variants, disrupting the balance between pro- and anti-inflammatory signals [39]. Additionally, mutations in *AP1S3*—a gene encoding a subunit of the adaptor protein complex 1 (AP-1)—may impair vesicular trafficking and Toll-like receptor 3 function in keratinocytes, further contributing to disease pathogenesis [40].

In summary, whereas psoriasis vulgaris is primarily driven by a dominant IL-23/Th17 axis, PPP exhibits a more complex inflammatory profile that integrates IL-36, IL-23/Th17, Th2, and IL-8/neutrophil pathways, compounded by smoking-related factors and a distinct Th2-skewed genetic background. This unique inflammatory milieu underscores the need for tailored therapeutic strategies and the development of targeted therapies to optimize patient outcomes.

## 3. Mechanism of Action of IL-23 Inhibitors

IL-17 cytokine family members—particularly IL-17A and IL-17F—are produced by various immune cells (including CD4^+^ and CD8^+^ T cells, γδ T cells, and several innate immune cells) in response to stimuli such as IL-1β and IL-23 [41]. These cytokines are essential for neutrophil recruitment, antimicrobial peptide production, and barrier integrity; however, when dysregulated, they contribute to the immunopathology observed in infections and autoimmune conditions [41]. For instance, IL-17 inhibitors may exacerbate or even induce inflammatory bowel disease and mucosal candidiasis [42]. This underscores the need for alternative strategies that modulate IL-17–mediated pathology without compromising host defense.

One such alternative is IL-23 inhibitors, which selectively target the IL-23/Th17 axis—a pivotal driver of inflammation in immune-mediated diseases like PPP. IL-23 is a heterodimer composed of a unique p19 subunit and a shared p40 subunit with IL-12, and it is critical for the differentiation, survival, and expansion of Th17 cells. By binding to the p19 subunit, agents such as guselkumab, risankizumab, and tildrakizumab block the interaction between IL-23 and its receptor on Th17 cells [43]. This blockade reduces the production of downstream pro-inflammatory cytokines of IL-17A, IL-17F, and IL-22, thereby mitigating keratinocyte hyperproliferation, neutrophil recruitment, and chronic inflammation in PPP [41].

In contrast to IL-17A inhibitors (e.g., secukinumab, ixekizumab) and the IL-17A/F inhibitor (e.g., bimekizumab), which directly neutralize IL-17 from all cellular sources and provide rapid symptomatic relief but may exert broader immunosuppressive effects, IL-23 inhibitors act upstream by selectively modulating the IL-23–dependent activation of pathogenic Th17 cells. This upstream action helps preserve IL-12–mediated responses that are essential for intracellular pathogen defense, potentially lowering the risk of opportunistic infections (such as mucocutaneous candidiasis) and reducing the likelihood of inflammatory bowel disease exacerbation [41,44]. However, because IL-23 inhibitors indirectly reduce IL-17 production, they tend to have a slower onset of action—a possible limitation in managing the acute, debilitating symptoms of PPP. Moreover, the IL-23–independent production of IL-17A and IL-17F by innate and innate-like lymphocytes results in distinct efficacy and safety profiles between IL-17 and IL-23 inhibitors [45].

**Figure 1:**
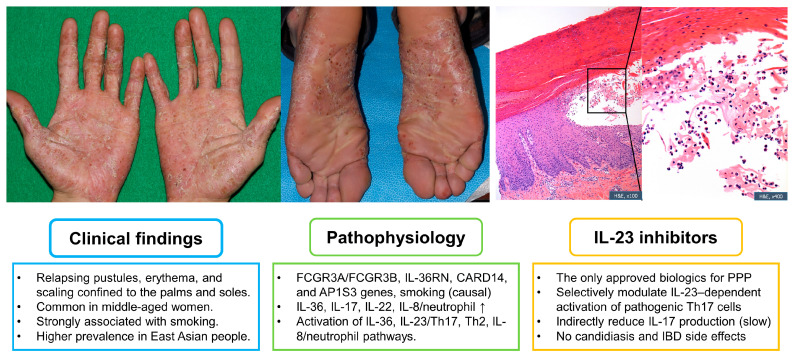
Overview of the example case, including histological findings, a summary of clinical findings, the pathophysiology of PPP, and the mechanism of action of IL-23 inhibitors. The example case describes a 39-year-old male presenting with pruritic, diffuse, hyperkeratotic plaques accompanied by pustules on both palms and soles for two months. A potassium hydroxide test yielded negative results. A skin biopsy revealed intraepidermal pustules predominantly filled with neutrophils, with the surrounding epidermis displaying psoriasiform dermatitis.

Taken together, while both classes target key components of the inflammatory cascade, IL-23 inhibitors may offer a more favorable long-term safety profile for chronic conditions such as PPP. Figure 1 illustrates the example case with histological findings, summary of clinical findings, pathophysiology of PPP, and the mechanism of action of IL-23 inhibitors.

## 4. Clinical Evidence for IL-23 Inhibitors in PPP

### 4.1. Guselkumab

A 24-week, phase 2, double-blind, randomized, placebo-controlled proof-of-concept study evaluated the efficacy and safety of guselkumab in 49 Japanese patients with moderate-to-severe PPP. Patients received guselkumab (200 mg) or placebo at weeks 0 and 4, with outcomes assessed through week 24. At week 16, guselkumab produced a significant improvement in the PPP Severity Index (PPSI) (least squares mean difference: −1.5, *p* = 0.03) and a significant reduction in the PPP Area and Severity Index (PPPASI) (least squares mean difference: −5.65, *p* = 0.009), with 39.2% more patients achieving a 50% reduction in PPPASI compared to placebo. Additionally, serum levels of IL-17A and IL-17F were markedly reduced following treatment [46].

In a separate phase 2, multicenter, single-arm trial involving 50 European patients with moderate-to-severe PPP, patients received 100 mg of guselkumab subcutaneously at weeks 0, 4, and 12, then every 8 weeks until week 24. The study met its primary endpoint with a median PPPASI reduction of 59.6% at week 24 (*p* < 0.001), and the response rates for PPPASI-50 and PPPASI-75 were 66.0% and 34.0%, respectively, suggesting efficacy in European populations [47].

A subsequent phase 3, 52-week, double-blind, randomized, placebo-controlled trial in 159 Japanese patients with moderate-to-severe PPP unresponsive to conventional therapies compared two guselkumab doses (100 mg and 200 mg) with a placebo. At week 16, the 100 mg group exhibited a significantly greater reduction in PPPASI (−15.3 vs. −7.6; *p* < 0.001), with 57.4% achieving PPPASI-50 versus 34.0% in the placebo group (*p* = 0.02). Although the 200 mg group showed numerical improvements, these did not reach statistical significance. Clinical benefits were sustained through week 52, as reflected in improved PPSI scores [16].

A long-term extension of the phase 3 trial assessed guselkumab’s sustained efficacy and safety through week 84 in Japanese patients. Those initially treated with guselkumab (100 mg or 200 mg) or re-randomized from placebo showed mean improvements of approximately 79% in PPPASI and 66% in PPSI. Better long-term responses were associated with the absence of prior phototherapy, non-use of non-biologic systemic therapy, and non-smoking status. Guselkumab was well tolerated, with most treatment-emergent adverse events (TEAEs) being mild to moderate (≈88%) and a low incidence of serious TEAEs (7.6%) [48].

A biomarker study investigated guselkumab’s effects on serum cytokine levels over 72 weeks. Baseline levels of IL-17A, IL-17F, and IL-22 were elevated in PPP patients and correlated with disease severity. Treatment with guselkumab significantly reduced these cytokines (maximal reductions at week 72, *p* < 0.01), and lower cytokine levels were linked with improved PPPASI scores. Notably, both the 100 mg and 200 mg doses produced similar pharmacodynamic effects, suggesting no additional benefit at the higher dose [49].

A retrospective single-center study in 17 Korean patients with refractory PPP evaluated guselkumab 100 mg. With a median baseline PPPASI of 12.2 (range: 4.2–31.2), 82.4% achieved PPPASI-50 and 47.1% reached PPPASI-75 by study end. Concurrent systemic therapies (acitretin or cyclosporine) were used in 58.8% of patients. Only two mild injection site reactions were reported, and no serious adverse events occurred during treatment (up to 118 weeks). A lower body mass index (BMI) was associated with better responses, while factors such as gender, age, baseline PPPASI, disease duration, concomitant therapy, smoking status, and history of tonsillitis did not significantly affect outcomes. These findings are consistent with the Japanese phase 3 trial data, supporting guselkumab’s efficacy and tolerability in Korean patients [50].

**Figure 2:**
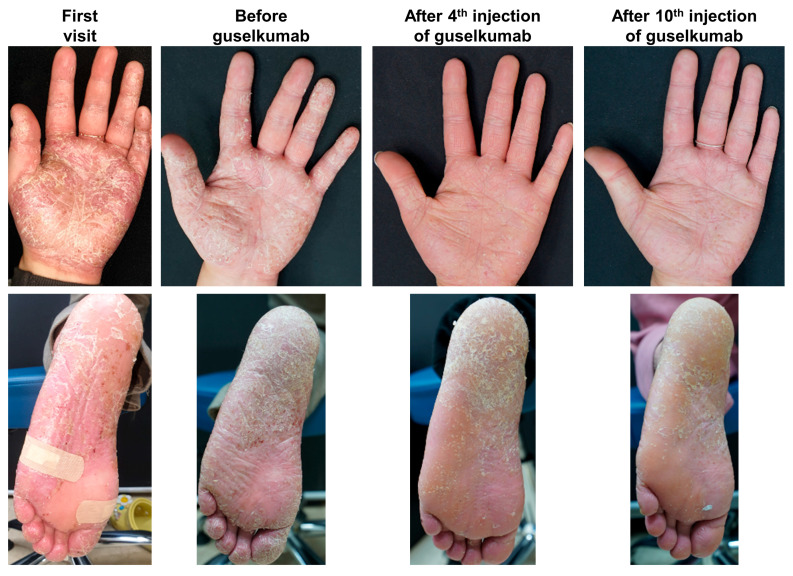
Case presentation of PPP treated with guselkumab. This case features a 24-year-old female with painful pustular eruptions on her palms and soles. Skin biopsy showed intraepidermal pustules predominantly composed of neutrophils, accompanied by psoriasiform epidermal hyperplasia, which was consistent with the clinical diagnosis of PPP. The patient showed minimal improvement after one year of treatment with acitretin (20 mg daily) and cyclosporin (150 mg daily). Consequently, guselkumab treatment was initiated. Following the fourth injection of guselkumab (week 28), the lesions demonstrated marked improvement. This positive outcome persisted after the tenth injection (week 40) without significant side effects.

We present a case report of a PPP patient treated with guselkumab. A 24-year-old female with painful pustular eruptions on her palms and soles had shown minimal improvement with acitretin (20 mg daily) and cyclosporin (150 mg daily) over one year. Skin biopsy showed intraepidermal pustules predominantly composed of neutrophils, accompanied by psoriasiform epidermal hyperplasia, which was consistent with the clinical diagnosis of PPP. Initiation of guselkumab led to marked improvement after the fourth injection (week 28), and the positive response was sustained after the tenth injection (week 40) (Figure 2).

### 4.2. Risankizumab

The JumPPP study, a phase 3, multicenter, randomized, placebo-controlled, double-blind trial, assessed risankizumab in 119 Japanese patients with PPP. Patients were randomized to receive 150 mg risankizumab or placebo for 16 weeks, followed by an open-label phase where all participants received risankizumab. At week 16, a significantly higher proportion of risankizumab-treated patients achieved PPPASI-50 compared to placebo (41.0% vs. 24.1%; *p* < 0.05), although PPPASI-75 rates were similar between groups (13.1% vs. 15.5%; *p* = 0.74). By week 68, 87.0% of patients achieved PPPASI-50 and 57.4% reached PPPASI-75, with a safety profile consistent with previous studies and no new safety concerns identified. These findings indicate that long-term risankizumab treatment provides sustained efficacy and is well tolerated in Japanese patients with PPP [17].

### 4.3. Others

A retrospective study examined the use of anti-IL-23p19 monoclonal antibodies in 16 European patients with PPP at two German tertiary dermatological centers. Treatments included guselkumab (n = 12), risankizumab (n = 3), and tildrakizumab (n = 1), administered at doses approved for plaque-type psoriasis. The median treatment duration was 12.5 months. At 12 weeks, 56.3% of patients achieved PPPASI-50 and 31.3% reached PPPASI-75; by 12 months, these rates increased to 62.5% and 43.8%, respectively. Five patients (31.3%) discontinued treatment due to insufficient clinical improvement. No significant adverse effects were observed, and factors such as disease duration, smoking status, and concomitant plaque-type psoriasis did not significantly influence therapeutic outcomes. Although the sample size was limited, this study suggests that IL-23 inhibitors may be beneficial in real-world European settings [51].

### 4.4. Summary

Collectively, the current evidence indicates that IL-23 inhibitors represent a promising therapeutic strategy for PPP, with favorable efficacy and safety profiles. Guselkumab has the most robust supporting data, followed by risankizumab, while evidence for tildrakizumab remains limited. Clinical improvements with IL-23 inhibitors may take up to 24 weeks to reach maximal effect, emphasizing the need to educate patients on the expected timeline for therapeutic response. Further large-scale, long-term studies are warranted to validate these findings and optimize IL-23 inhibitor use across diverse patient populations.

## 5. Other Biologics for PPP

### 5.1. IL-12/23 Inhibitors (Ustekinumab)

In a randomized trial, patients with PPP received either ustekinumab (45/90 mg; 45 mg for patients weighing less than 100 kg and 90 mg for those weighing 100 kg or more) or placebo. In the ustekinumab group, patients received the active drug at weeks 0, 4, and 16, followed by placebo at week 20; the placebo group received placebo at weeks 0 and 4, then ustekinumab at weeks 16 and 20. The primary endpoint was the proportion of patients achieving a PPPASI-50 response at week 16. No statistically significant difference in PPPASI-50 response was observed between the ustekinumab and placebo groups (*p* = 1.000) [24].

### 5.2. IL-17 Inhibitors (Secukinumab, Brodalumab)

The 2PRECISE trial, a phase 3 multicenter, randomized, double-blind, placebo-controlled study, evaluated secukinumab in 204 patients with moderate-to-severe PPP. Patients were randomized to receive either secukinumab 300 mg (n = 68), secukinumab 150 mg (n = 69), or placebo (n = 67) over 52 weeks. Although the primary endpoint—PPPASI-75 at week 16 using a 2.5% significance threshold—was not met, 26.6% of patients receiving secukinumab 300 mg achieved PPPASI-75 compared to 14.1% in the placebo group at week 16 (*p* = 0.0411; OR, 2.26; 95% CI, 1.04–4.89). By week 52, 41.8% of patients on secukinumab 300 mg reached PPPASI-75. No unexpected safety concerns were observed, suggesting that secukinumab may offer clinical benefits, particularly with long-term treatment [52].

In a separate phase 3, randomized, double-blind, placebo-controlled trial involving 126 Japanese patients with moderate-to-severe PPP, brodalumab—an anti-IL-17 receptor A monoclonal antibody—was evaluated. Patients were randomized 1:1 to receive brodalumab 210 mg (administered at baseline, weeks 1 and 2, then every 2 weeks until week 16) or placebo. Brodalumab significantly improved total PPPASI scores at week 16 (least squares mean difference: −5.29; *p* = 0.0049) and yielded higher PPPASI-50/75/90 response rates (54.0%, 36.0%, and 16.0%, respectively) compared to placebo. Common treatment-emergent adverse events included otitis externa, folliculitis, and nasopharyngitis, mostly of mild-to-moderate severity [53].

### 5.3. IL-36 Inhibitors (Spesolimab)

A phase 2b trial evaluated spesolimab, an anti-IL-36 receptor antibody, in 155 patients with moderate-to-severe PPP. Patients were randomized to receive various doses of spesolimab or placebo. Although the primary endpoint—percentage change in PPPASI at week 16—was not met (with mean differences ranging from −14.6% to −5.3% between spesolimab and placebo), secondary endpoints were encouraging. At week 16, 21.1% of spesolimab-treated patients achieved a physician’s global assessment (PGA) score of 0/1 compared to 4.7% in the placebo group; by week 52, this proportion increased to 54.1%. Notably, significant improvements were observed in non-Asian patients, and the safety profile was comparable to the placebo despite a higher incidence of injection-site reactions [54].

### 5.4. IL-1 Inhibitors (Anakinra)

In a phase 4 trial, anakinra—an IL-1 receptor antagonist—was administered via daily subcutaneous injections for 8 weeks in 64 adults with PPP. The study did not demonstrate a significant change in PPPASI at week 8 (mean difference: −1.65; *p* = 0.300), and secondary outcomes, including pustule counts and quality-of-life indices, failed to show superiority over placebo. Although 41% of anakinra-treated patients perceived the treatment as beneficial, the high rate of injection-site reactions (61% vs. 3% in the placebo group) and lack of significant efficacy suggest that IL-1 blockade is not a viable strategy for managing PPP [55].

### 5.5. Dual IL-17A/F Inhibitors (Bimekizumab)

Bimekizumab, a dual IL-17A and IL-17F inhibitor, has shown superior efficacy in psoriasis compared with existing IL-17 inhibitors [56]. A retrospective case series in 21 adults with PPP from seven French tertiary dermatology centers found that most patients—who had failed prior systemic treatments—received bimekizumab for at least three months. Remarkably, 81% (17 of 21) achieved complete clearance (IGA score of 0) within 1–4 months. Adverse events were minimal; candidiasis occurred in 19% of patients but was managed successfully without discontinuing therapy [57]. These findings suggest that bimekizumab may be a highly effective option for PPP, with the additional blockade of IL-17F potentially enhancing its therapeutic benefits compared to IL-17A inhibitors alone. Further evaluation in larger, controlled trials is warranted.

## 6. Small Molecules for PPP

### 6.1. Apremilast

Apremilast is an oral phosphodiesterase-4 (PDE4) inhibitor approved for moderate-to-severe plaque psoriasis and psoriatic arthritis. Among small-molecule inhibitors, it has the most robust evidence in PPP, with two pivotal randomized clinical trials supporting its effectiveness in improving disease severity and quality of life [58,59]. Several case series have also highlighted its therapeutic potential in PPP [60,61,62].

In a phase 2, randomized, double-blind, placebo-controlled study involving 90 Japanese patients with PPP unresponsive to topical treatments, 30 mg apremilast administered twice daily significantly outperformed placebo at week 16, with a higher proportion of patients achieving PPPASI-50 (*p* = 0.0003). Secondary endpoints—including improvements in total PPPASI score, PPSI, and patient-reported pruritus and pain—also reached significance, with benefits sustained through week 32. Common adverse events included diarrhea, abdominal discomfort, headache, and nausea, with no new safety concerns identified [58].

The APLANTUS study, a phase 2 single-arm multicenter trial involving 21 subjects with moderate-to-severe PPP, conducted over 20 weeks, demonstrated a median PPPASI reduction of 57.1% (*p* < 0.001). Additionally, 61.9% of patients achieved at least a 50% improvement in PPPASI, and 76.2% experienced a significant reduction in pustule count. Quality of life improved notably, as reflected by a decrease in median DLQI score from 8.5 to 2.0 (*p* = 0.030). Apremilast was well tolerated, with no serious adverse events reported [59].

A recent phase 3, randomized, double-blind, placebo-controlled trial evaluated the efficacy and safety of apremilast in 176 Japanese patients with moderate-to-severe PPP who had an inadequate response to topical therapies. Patients were randomized 1:1 to receive either apremilast or placebo for 16 weeks, followed by an open-label extension with apremilast through week 52. At week 16, 68% of patients treated with apremilast achieved PPPASI-50, compared to 35% in the placebo group (*p* < 0.0001). Improvements were sustained through week 52. The most common adverse events in the apremilast group included diarrhea (19%), soft stools (17%), headache (11%), and nausea (11%), with no serious or fatal events reported. This study confirms that apremilast is an effective and well-tolerated treatment option for PPP in Japanese patients [63].

### 6.2. Others

Upadacitinib, a selective Janus kinase 1 (JAK1) inhibitor approved for atopic dermatitis and psoriatic arthritis, has shown potential benefits in PPP through several case reports [64,65,66,67]. Similarly, deucravacitinib, an allosteric selective tyrosine kinase 2 (TYK2) inhibitor recently approved for psoriasis, has demonstrated potential in a small case series involving five PPP patients [68,69].

### 6.3. Summary

Collectively, small-molecule inhibitors offer a promising alternative to biologics in PPP treatment, potentially providing greater convenience and broader immunomodulatory effects. However, apart from apremilast—supported by phase 2 randomized controlled trials—the evidence for other small molecules in PPP is primarily derived from limited case series. These findings underscore the need for further large-scale, controlled trials to confirm their efficacy and long-term safety profiles.

## 7. Limitations and Future Directions

The most significant limitation of our review is the lack of direct head-to-head clinical trials comparing different biologics and small-molecule inhibitors in patients with PPP. Due to the relatively lower prevalence of PPP compared with psoriasis vulgaris, there is a scarcity of clinical evidence regarding treatment modalities for PPP, which has led to limited treatment options. Furthermore, current management of PPP tends to follow treatment options for psoriasis vulgaris, and specific guidelines for its management are lacking. Additional head-to-head, randomized clinical trials are crucial for drawing definitive conclusions about optimal treatment strategies.

Most clinical trials have been conducted in Caucasian or Japanese patients with PPP, which hinders the extrapolation of these results to the general population. The higher prevalence of PPP in East Asian people suggests potential clinical and biological differences between ethnicities that warrant further investigation through dedicated clinical trials in diverse populations.

The long-term efficacy and safety profile of IL-23 inhibitors and other therapies in PPP is not yet fully established. Due to the short duration of biological agent use in PPP patients and the small number of relevant randomized controlled trials, treatment durations and sample sizes have not been sufficiently large to draw conclusive remarks. For example, the study with the longest treatment period in PPP was a long-term extension study of guselkumab, which demonstrated sustained efficacy and safety over 1.5 years in a randomized phase 3 trial [48]. Further long-term extension studies are needed to assess the potential long-term benefits and risks of IL-23 inhibitors and other therapies in PPP

Due to its lower prevalence and the nature of the disease, PPP is widely acknowledged as a diagnostic and therapeutic challenge for many dermatologists. Acral regions have a thicker stratum corneum and require longer tissue regeneration times, resulting in delayed treatment responses. Consequently, maximal treatment response may require an extended period to demonstrate significant effectiveness. Additionally, the continuous exposure of acral regions to various external environmental factors may lead to variable treatment responses. PPASI-based outcomes may not be sufficiently sensitive to detect changes in diseases of limited extent such as PPP. These factors may also contribute to the high and variable placebo response rates observed in PPP clinical trials, hindering their success and contributing to the limited number of ongoing studies

## 8. Discussion

Biologics and small-molecule inhibitors targeting specific cytokines have transformed the treatment landscape for immune-mediated diseases such as psoriasis vulgaris and PPP. This review comprehensively examined the current evidence for IL-23 inhibitors, other biologics, and small-molecule inhibitors in PPP, focusing on IL-23 inhibitors as a promising treatment option with a favorable safety profile. We summarized key clinical trials of IL-23 inhibitors and other therapies in PPP (Table 1 and Table 2). Among the available agents, guselkumab is supported by the most robust data, followed by risankizumab, while evidence for tildrakizumab remains limited. Large-scale, controlled trials are essential to confirm their long-term efficacy and safety and refine treatment protocols for PPP.

IL-23 inhibitors are indicated for the treatment of PPP in adult patients who have not adequately responded to conventional therapies. Contraindications are limited to clinically significant active infections—including active tuberculosis—and known hypersensitivity to the active substance or any of its excipients. For patients with special cases, such as chronic viral infections (e.g., hepatitis B or C), malignancies, or latent tuberculosis, thorough screening before treatment and close monitoring during treatment with specialist consultation are recommended. Patients should also be closely monitored throughout treatment, with consultation from specialists as needed. Preemptive therapy, such as antiviral therapy for hepatitis or antituberculosis treatment for latent tuberculosis, may be considered, as needed.

For patients previously treated with biologics without adequate response, biologic switching may be considered. However, limited evidence exists regarding the optimal approach to biologic switching, both in PPP and in more common forms of psoriasis, such as plaque psoriasis. Clinicians should carefully consider biologic switching to or from IL-23 inhibitors, as this decision should be made with caution due to the limited evidence available regarding its efficacy in such cases.

Mixed results have been observed with IL-17 inhibitors, such as secukinumab and brodalumab, which have shown promise despite not consistently meeting primary endpoints in clinical trials. In contrast, IL-1 and IL-36 blockade have yet to demonstrate clear efficacy in PPP treatment. Emerging agents like bimekizumab, which inhibits both IL-17A and IL-17F, may offer additional benefits over existing therapies and warrant further evaluation.

Among emerging small-molecule therapies, apremilast has demonstrated the most promising efficacy in PPP from a recent phase 3 clinical trial. Although indirect comparisons should be interpreted cautiously, a phase 3 trial of apremilast in Japanese patients reported a PPPASI-50 response of 67.8% at week 16 (vs. 35.3% with placebo), which was numerically higher than those observed in phase 3 trials of guselkumab (57.4% vs. 34.0%) and risankizumab (41.0% vs. 24.1%). These differences may be partly explained by variations in the study design, patient populations, or the relatively slower onset of action associated with IL-23 inhibitors.

Nevertheless, IL-23 inhibitors are still often preferred in clinical practice, despite possibly lower response rates and higher costs. This may reflect apremilast’s off-label status for PPP and its association with a relatively higher incidence of common gastrointestinal side effects, such as diarrhea and nausea. Given the promising results of the recent phase 3 trial in Japan, there is potential for apremilast to receive approval for PPP treatment in the future. In addition, direct head-to-head trials are warranted to clarify comparative efficacy and safety profiles between apremilast and IL-23 inhibitors.

## 9. Conclusions

PPP is a rare disease, which is particularly prevalent in East Asian populations, and its pathogenesis involves complex immune dysregulation, with activation of the IL-23/Th17 axis. This review focused on the mechanisms of action, efficacy, and safety profiles of IL-23 inhibitors, which are the only currently approved advanced treatment option for PPP patients who have not responded adequately to conventional therapies. Additionally, emerging data suggest that apremilast may also be a promising agent for PPP treatment in the future. However, research on the treatment of PPP remains limited, and further studies and clinical trials are needed to better understand the optimal treatment strategies.

## Figures and Tables

**Table 1:** Summary of efficacy results from the key clinical trials for IL-23 inhibitors in PPP.

Ref.	Patients (n)	Intervention	Main Efficacy Results
[46]	24-week phase 2 RCT in Japanese patients with moderate-to-severe PPP (n = 49)	Guselkumab 200 mg or placebo at W0 and 4	PPPASI improvement at 16 weeks was significantly higher in guselkumab vs. placebo (*p* = 0.009). PPPASI-50 at 16 weeks was significantly higher in guselkumab vs. placebo (*p* = 0.009).
[47]	24-week single-arm, phase 2 study in Caucasian patients with moderate-to-severe PPP (n = 50)	Guselkumab 100 mg at week 0, 4, 12, and 20	Median PPPASI reduction by 59.6% at week 24 compared to baseline (*p* < 0.001) PPPASI-50 and PPPASI-75 at week 24: 66.0% and 34.0%.
[16]	60-week phase 3 RCT in Japanese patients with moderate-to-severe PPP (n = 159)	Guselkumab 100 or 200 mg or placebo at week 0, 4, 12; all guselkumab thereafter every 8 weeks	Both guselkumab groups demonstrated significant PPPASI improvement vs. placebo (*p* < 0.001) The guselkumab 100 mg group (57.4%) achieved a significantly higher PPPASI-50 response at week 16 vs. placebo (34.0%; *p* = 0.02); however, it was not significant for the guselkumab 200 mg group (36.5%, *p* = 0.78). The efficacy endpoint improved consistently through week 52
[48]	82-week long-term extension of the phase 3 RCT in a Japanese patient with moderate-to-severe PPP (n = 133)	After a 60-week trial, the patients were followed up until 84 weeks	The mean improvement in the guselkumab groups from baseline in the PPPASI at week 84 was ~79%.
[50]	A retrospective, single-center RWE study in Korean patients with moderate-to-severe PPP (n = 17)	More than 4 cycles of guselkumab 100 mg 58.8% used concurrent systemic therapy with acitretin or cyclosporin	At week 28, PPPASI-50 (82.4%) and PPPASI-75 (47.1%).
[17]	68-week phase 3 RCT in Japanese patients with moderate-to-severe PPP (n = 119)	Risankizumab 150 mg or placebo at week 0, 4, 16; all risankizumab thereafter every 12 weeks	PPPASI improvement at week 16 was significantly higher in risankizumab vs. placebo (*p* < 0.05) At week 16, PPPASI-50 was significantly higher in risankizumab (41.0%) vs. placebo (24.1%) (*p* < 0.05), but not for PPPASI-75 (13.1% vs. 15.5%, *p* = 0.74) At week 68, PPPASI-50 (87.0%; 90.9%) and PPPASI-75 (57.4%; 69.1%) of patients in the continuous risankizumab;placebo-to-risankizumab groups, respectively.
[51]	A retrospective, two-center RWE study in German patients with moderate-to-severe PPP (n = 16)	Guselkumab (n = 12), Risankizuamb (n = 3), Tildrakizumab (n = 1) as per label in psoriasis	At week 12, PPPASI-50 (56.3%) and PPPASI-75 (25.0%) At week 48, PPPASI-50 (62.5%) and PPPASI-75 (43.8%)

PPP (Palmoplantar pustulosis); PPPASI (Palmoplantar pustulosis area and severity index); RCT (randomized controlled trial); RWE (Real-world evidence).

**Table 2:** Summary of efficacy results from the key clinical trials for other biologics and small molecular inhibitors in PPP.

Ref.	Patients (n)	Intervention	Main Efficacy Results
[24]	16-week RCT in Caucasian patients with PPP (n = 33)	Ustekinumab 45/90 mg or placebo at week 0 4, and 16	Not significant difference in PPPASI-50 at 16 week in ustekinumab vs. placebo (*p* = 1.000).
[52]	52-week phase 3 RCT in Caucasian patients with moderate-to-severe PPP (n = 50)	Secukinumab 150 or 300 mg or placebo SC once weekly at week 1, 2 and 3, and per 4 weeks	At 16 weeks, PPPASI-75 with secukinumab 300 mg (26.6%, *p* = 0.041) vs. placebo At 16 weeks, PPPASI-75 with secukinumab 150 mg (17.5%, *p* = 0.572) vs. placebo At 52 week, PPPASI-75 41.8% for secukinumab 300 mg
[53]	16-week phase 3 RCT in Japanese patients with moderate-to-severe PPP followed by a 52-week, open-label extension period (n = 112)	Brodalumab 210 mg or placebo SC at weeks 0, 1, 2, and per 2 weeks	At 16 weeks, PPPASI’s improvement was significantly higher with brodalumab vs. placebo (*p* = 0.0049). At 16 weeks, PPPASI-50/75/90: 54% vs. 24.2%/36.0% vs. 8.1%/16.0% vs. 0.0% (brodalumab vs. placebo)
[54]	52-week phase 2 RCT in patients with moderate-to-severe PPP (n = 152)	Spesolimab (various) ^1^ or placebo SC per 4 weeks; thereafter, spesolimab per 4 weeks at week 16	Mean differences for spesolimab vs. placebo: ranged from—14.6% to—5.3%; none reached significance. At 16 weeks, PGA 0/1: 21.1% and 4.7% of patients in the spesolimab and placebo groups. At 52 weeks, PGA 0/1: 54.1% and 27.9% of patients in the spesolimab and placebo-switch patients.
[55]	8-week phase 4 RCT in patients with PPP requiring systemic therapy (n = 64)	Daily anakinra or placebo SC for 8 weeks	Mean PPPASI difference at week 8 for anakinra versus placebo: −1.65 (*p* = 0.300). Mean PPPASI-50/90 difference at week 8 for anakinra vs. placebo: 2.30 (*p* = 0.287)/3.80 (*p* = 0.285).
[58]	32-week phase 2 RCT in Japanese patients with PPP requiring systemic therapy (n = 90)	Oral apremilast 30 mg twice daily or placebo for 16 weeks; thereafter, apremilast until 32 weeks	PPPASI-50 response at week 16 was significantly higher with apremilast vs. placebo (*p* = 0.0003). PPPASI improvement at week 16 was significantly higher with apremilast vs. placebo (*p* = 0.0013). Improvements were sustained through week 32 with apremilast.
[59]	20-week phase 2 single-arm study in Japanese patients with moderate-to-severe PPP (n = 21)	Oral apremilast 30 mg twice daily until 20 weeks	PPPASI at week 20 showed a median reduction of 57.1% (*p* < 0.001) PPASI-50 response at week 20 was 61.9%
[63]	52-week phase 3 RCT in Japanese patients with moderate-to-severe PPP (n = 176)	Oral apremilast 30 mg twice daily or placebo for 16 weeks; thereafter, apremilast until 52 weeks	PPPASI-50 response at week 16 was significantly higher with apremilast (68%) vs. placebo (35%) (*p* = 0.0003). PPPASI improvement at week 16 was significantly higher with apremilast vs. placebo (*p* < 0.0001). Improvements were sustained through week 52 with apremilast.

^1^ Spesolimab 3000 mg to W4 then 600 mg Q4W, spesolimab 3000 mg to W4 then 300 mg Q4W, spesolimab 1500 mg to W4 then 600 mg Q4W, spesolimab 1500 mg to W4, 300 mg Q4W to Q8W, or placebo switching to spesolimab 600 mg Q4W at W16. PGA (physician’s global assessment); PPP (Palmoplantar pustulosis); PPPASI (Palmoplantar pustulosis area and severity index); vs. (versus).
